# Vector Form of Symmetry Degree

**DOI:** 10.1038/s41598-017-13405-0

**Published:** 2017-10-11

**Authors:** G. H. Dong, Z. W. Zhang, C. P. Sun, Z. R. Gong

**Affiliations:** 1 College of Physics and Energy, Shenzhen University, Shenzhen, 518060 P. R. China; 2 Beijing Computational Science Research Center, Beijing, 100084 China

## Abstract

Symmetry degree is utilized to characterize the asymmetry of a physical system with respect to a symmetry group. The scalar form of symmetry degree (SSD) based on Frobenius-norm has been introduced recently to present a quantitative description of symmetry. Here we present the vector form of the symmetry degree (VSD) which possesses more advantages than the SSD. Mathematically, the dimension of VSD is defined as the conjugacy class number of the symmetry group, the square length of the VSD gives rise to the SSD and the direction of VSD is determined by the orders of the conjugacy classes. The merits of applying VSD both for finite and infinite symmetry groups include the additional information of broken symmetry operators with single symmetry breaking perturbation, and the capability of distinguishing distinct symmetry breaking perturbations which exactly give rise to degenerate SSD. Additionally, the VSD for physical systems under symmetry breaking perturbations can be regarded as a projection of the initial VSD without any symmetry breaking perturbations, which can be described by an evolution equation. There are the same advantages by applying VSD for the accidental degeneracy and spontaneous symmetry breaking.

## Introduction

Symmetry is always an intriguing topic of modern physics, which plays a crucial role in understanding the fundamental interactions and the structures of physical systems ranging from micro to macro levels^1,2^. The application of the symmetry especially the spontaneous symmetry breaking has inspired many fields of physics, such as particle physics^3–8^, condensed matter physics^9–12^, physical chemistry^13–16^, biological physics^17,18^ and quantum information^19,20^. Mathematically, symmetry refers to that a system is invariant under some specific transformations. This dichotomous definition indicates a system either possesses or lacks of a symmetry. However, for a physical symmetric system with slightly symmetry breaking, it still can be approximately treated under the original symmetry. For instance, the crystal structure of bilayer transition metal chalcogenide possesses *D*_3*h*_ symmetry and an out-of-plane electric field is usually applied which breaks the symmetry into its subgroup *C*_3*V*_. However, in most of cases the stark effect resulting from the electric field is sufficiently weak, *D*_3*h*_ symmetry is still applied to obtain the energy spectrum and the electronic dynamics in such system. In this sense, the symmetry needs a continuous quantitative description for physical systems.

A scalar form of continuous symmetry degree (SSD) has already been proposed in ref.^21^. Since for a physical system all the symmetric transformations form a symmetry group, a symmetry breaking perturbation gives rise to a reduction of the symmetry group into its corresponding subgroup. Based on the irreducible representation of the symmetry group and the Frobenius norm^22^, the SSD ranging from zero to unity is more capable to identify various nature of symmetries such as symmetry breaking, accidental degeneracy and spontaneous symmetry breaking^23^, in contrast to the previous explorations based on the abstract concepts of fuzz set^24^ and transform information^25^. Recently, it was applied to the Frobenius norm-based measures for quantum coherence and asymmetry^26^.

Nevertheless, the SSD basically is an average of the Frobenius norm over all transformations in the symmetry group. More information of symmetry degree on an individual transformation is lost during this averaging calculation, which is supposed to reveal the delicate nature of symmetry. To avoid this artificial missing, we propose the vector form of the symmetry degree (VSD) instead, which is obtained by dividing the SSD according to the conjugacy classes of the symmetry group. In this sense, the dimension of VSD exactly equals to the conjugacy class number of the symmetry group and the square length of the VSD gives rise to the SSD. It is easy to prove that the VSD inherits those good properties from SSD, such as vector length range from zero to unity, basis-independent, invariant under the zero-point energy shifting as well as the scaling transformation. One more important property for VSD is that the information of symmetry degree on individual transformations in the same conjuagcy class is stored into the component of the VSD, which is illustrated by the direction of the VSD. For example, for a symmetry breaking perturbation, the symmetry nature can be delicately described by identifying the disappearance of components of VSD. The other merit of applying VSD both for finite and infinite symmetry groups is the capability of distinguishing distinct symmetry breaking perturbations which exactly give rise to degenerate SSD. When the symmetry breaking perturbation increases, the trajectory of the VSD in the parameter space can be regarded as a projection process of the initial VSD. There are the same advantages by applying VSD for the accidental degenerate and spontaneous symmetry breaking systems. The accurate definition of the continuous quantitative description of the symmetry may shed light on symmetry related applications in various physics fields.

## Results

### Definition of Vector form of symmetry degree

We start from a physical system with Hamiltonian *H* and a symmetry group *G* with *n*_*g*_ transformations defined on the physical system’s Hilbert space. For any element *O* ∈ *G*, the deviation between *OHO* ^−1^ and *H* measures the asymmetry of physical system with respect to the set element *O*. Thus the SSD is defined as the average over *G*

1 $${\mathscr{S}}\,(G,H)=\frac{1}{4{|\mathop{H}\limits^{ \sim }|}^{2}{n}_{g}}\sum _{g\in G}{|\{R(g),\mathop{H}\limits^{ \sim }\}|}^{2},$$

where $|O|=\sqrt{Tr[{O}^{\dagger }O]}$ is the Frobenius norm measuring the fidelity of *O*, $R:g\to R(g)\in End({\mathscr{H}})$ is a *d*-dimensional representation of *g* ∈ *G*, $\mathop{H}\limits^{ \sim }=H-{d}^{-1}Tr\{H\}$ is a re-biased Hamiltonian, and $\{R(g),\mathop{H}\limits^{ \sim }\}=R(g)\mathop{H}\limits^{ \sim }+\mathop{H}\limits^{ \sim }R(g)$ is the anti-commutation operation.

Obviously, all the information about the symmetry nature with respect to an individual transformation is mixed during the averaging. However, the information of which exact transformation the Hamiltonian is symmetric or asymmetric with respect to is more important for the applications. In other hand, the symmetry group *G* can be divided into several conjugacy classes {*Gi*}. In one conjugacy class, any two elements *a*,*b*∈*G*_*i*_ are conjugate as *gag* ^−1^ = *b* for *g* ∈ *G*. This means the information of symmetry with respect to the conjugate transformations is not nesessary to be identified. In this sense, the VSD is defined based on the conjugacy classes as ${\bf{S}}(G,H)={\sum }_{i}{S}_{i}({G}_{i},H){{\bf{e}}}_{i},$ where

2 $${S}_{i}({G}_{i},H)=\frac{1}{2|\mathop{H}\limits^{ \sim }|\sqrt{{n}_{g}}}\sqrt{{\bar{{|\{R(g),\mathop{H}\limits^{ \sim }\}|}^{2}}}_{i}}$$

with

3 $${\bar{{|\{R(g),\mathop{H}\limits^{ \sim }\}|}^{2}}}_{i}=\sum _{g\in {G}_{i}}{|\{R(g),\mathop{H}\limits^{ \sim }\}|}^{2}.$$

Here, the summation is taken over the *i*-th conjugacy class, and **e**_*i*_ denotes the unit vector with respect to the *i*-th conjugacy class.

Obviously, the square length of the VSD ${|{\bf{S}}(G,H)|}^{2}={\sum }_{i}{S}_{i}^{2}({G}_{i},H)={\mathscr{S}}(G,H)$ gives rise to the SSD. Since the symmetry degree now is measured not only in a continuous description but also in a vector from, it actually introduces a geometric picture of the symmetry degree. More information of the symmetry nature can be illustrated by identifying the components of the VSD.

We will take a symmetry breaking system as an example. If *H*_0_ is symmetric under all transformations in group *G*, the ${\bf{S}}(G,{H}_{0})={\sum }_{i}{S}_{i}^{max}({G}_{i},{H}_{0}){{\bf{e}}}_{i}$ points to a definite direction with maximum unity length. An additional symmetry breaking perturbation *H*_1_ is applied to this physical system as *H* = *H*_0_+*λH*_1_ and the VSD is straightforwardly obtained as ${{\bf{S}}}^{\lambda }(G,H)={\sum }_{i}{S}_{i}^{\lambda }({G}_{i},H){{\bf{e}}}_{i}$ which possibly points to a different direction and its length is definitely smaller than unity. Here, *λ* quantifies the strength of the symmetry breaking perturbation. From the view of the vectors, **S** ^*λ*^ (*G*,*H*) can be regarded as a projection of ***S***(*G*, *H*_0_) as

4 $${{\bf{S}}}^{\lambda }\,(G,H)=\overleftrightarrow{{P}_{\lambda }}\,({H}_{1})\cdot {\bf{S}}(G,{H}_{0})$$

with a projection operator $\overleftrightarrow{{P}_{\lambda }}\,({H}_{1})={{\bf{S}}}^{\lambda }\,(G,H)\cdot {{\bf{S}}}^{\dagger }\,(G,{H}_{0})$. When *λ* varies from zero to infinity and the corresponding symmetry group changes respectively from group G to subgroup*G*′ due to *H*_1_, the VSD has a trajectory in the parameter space. In this sense, such trajectory is supposed to de described by an evolution equation as

5 $$\frac{d}{d\lambda }{{\bf{S}}}^{\lambda }\,(G,H)={\mathscr{S}}[{{\bf{S}}}^{\lambda }\,(G,H)-{{\bf{S}}}^{{\rm{\infty }}}\,(G,H)],$$

where the operator satisfies ${\mathscr{S}}[{\bf{0}}]=0$ in order to obtain the steady vector.

Another merit of the VSD is distinguishing distinct symmetry breaking perturbations which exactly give rise to degenerate SSD. Since it is very likely that the SSD gives two identical values for distinct symmetry breaking perturbations, which actually have different physical origins and result in asymmetry of the system for different transformations. For VSD as two vectors in the parameter space under such circumstance, even they possess the same length they can still be identified due to distinct directions. This will be demonstrated in details by the following examples, where VSD works well both for finite and infinite symmetry groups.

### Examples of physical systems for finite and infinite symmetry groups

**Figure 1:**
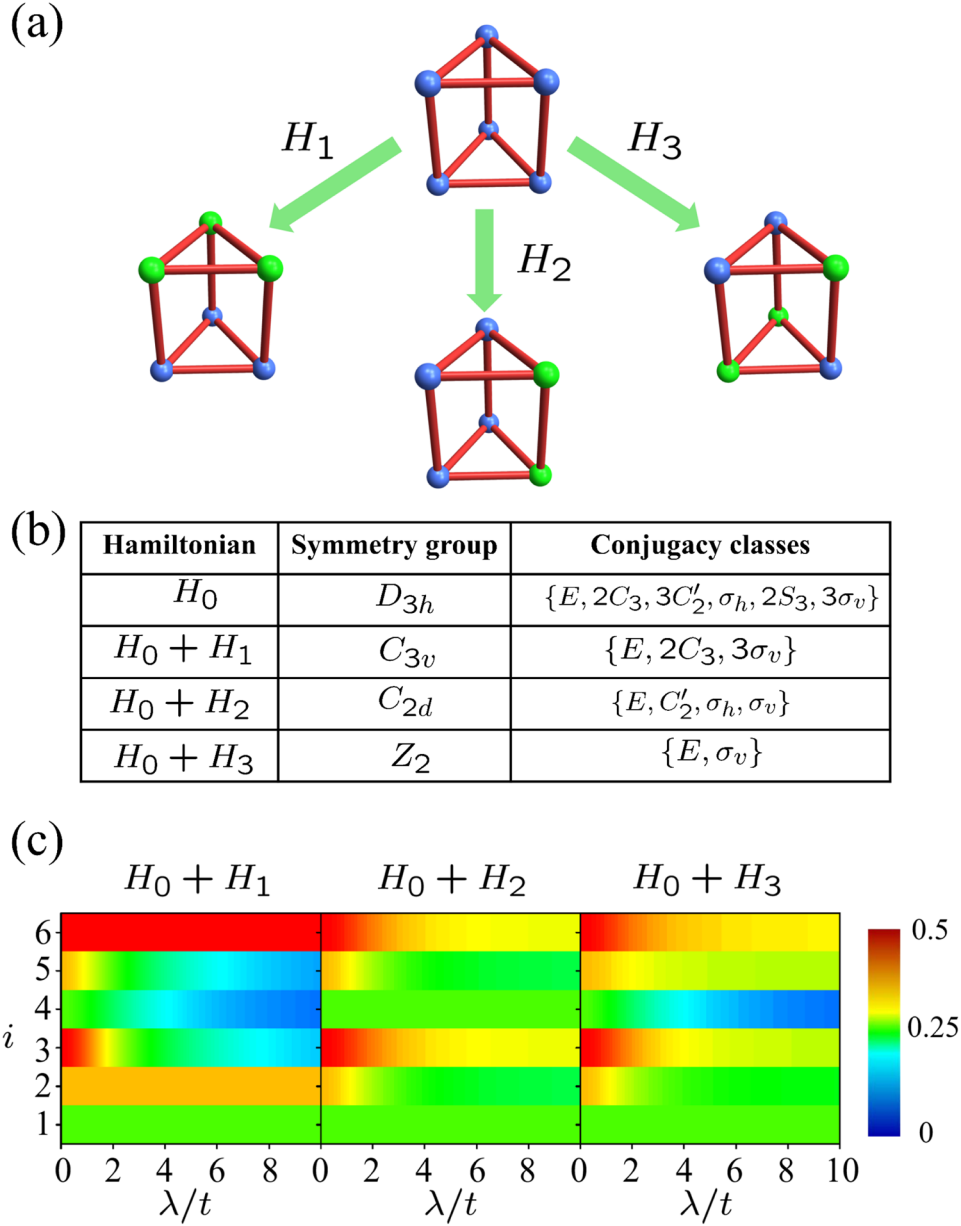
(**a**) Schematics of three kinds of symmetry breaking perturbations onto *D*_3*h*_. Here, blue spheres and bonds between them respectively denotes the particle and hopping between particles. The green spheres denotes the particles whose on-site energy have been changed bu symmetry breaking perturbations. (**b**) Table of Hamiltonian, the corresponding symmetry group and conjugacy classes. (**c**) Contourplot of the components of VSD *S*_*i*_ (*G*_*i*_, *H*) versus *i*− th conjugacy classes and the perturbation strength *λ*/*t* for three different symmetry breaking perturbations. Here, we have assumed ${\lambda }_{1}=\frac{2\sqrt{2}}{3}{\lambda }_{2}={\lambda }_{3}=\lambda$. Obviously, the VSDs for three distinct symmetry breaking perturbations differ from each other and thus VSD can be used to distinguish them.

First we consider a system with a finite symmetry group *D*_3*h*_ shown in Fig. 1(a). Such system with uniform triangular prism structure includes *Tc*_6_ *Cl*_6_^27^, bilayer graphene^28^, transition metal dichalcogenides^29,30^ and so on. They are described by a six-sites lattice Hamiltonian as

6 $${H}_{0}=\sum _{a=u,l}(\sum _{i=1}^{3}\varepsilon |i,a\rangle \langle i,a|+\sum _{i\ne j}{t}_{1}|i,a\rangle \langle j,a|+\sum _{i=1}^{3}{t}_{2}|i,a\rangle \langle i,{a}^{^{\prime} }|),$$

where $|i,a\rangle (i=1,2,3,a=u,l)$ is the single particle state with site *i* in the upper (*u*) or lower (*l*) layer occupied, the site energy *ε* and the intra-layer and inter-layer hopping strength *t*_1_ and *t*_2_ are site-independent for the uniform triangular prism geometry. The *D*_3*h*_ has six conjugacy classes as $\{E,\,2{C}_{3},\,3{C}_{2}^{^{\prime} },{\sigma }_{h},\,2{S}_{3},\,3{\sigma }_{v}\}$. Thus the maximum VSD is ${\rm{S}}({D}_{3h},{H}_{0})=\frac{1}{\sqrt{12}}(1,\sqrt{2},\sqrt{3},1,\sqrt{2},\sqrt{3})$.

Three different perturbations are taken into consideration as

7 $${H}_{1}=\sum _{i=1}^{3}{\lambda }_{1}\,|i,u\rangle \langle i,u|,$$

8 $${H}_{2}=\sum _{a=u,l}{\lambda }_{2}\,|1,a\rangle \langle 1,a|,$$

9 $${H}_{3}={\lambda }_{3}\,(|1,u\rangle \langle 1,u|+|2,l\rangle \langle 2,l|+|3,l\rangle \langle 3,l|),$$

which are depicted in Fig. 1(a). They respectively break *D*_3*h*_ into *C*_3*v*_,*C*_2*d*_ and *Z*_2_, whose corresponding conjugacy classes are presented in Fig. 1(b). According to the SSD definition in Eq. (1), the SSD for above three kinds of perturbations are straightforwardly obtained as ${{\mathscr{S}}}_{1}\,({D}_{3h},{H}_{0}+{H}_{1})=1-f\,({\lambda }_{1})/2$, ${{\mathscr{S}}}_{2}\,({D}_{3h},{H}_{0}+{H}_{2})=1-f(2\sqrt{2}{\lambda }_{2}/3)/2$, ${{\mathscr{S}}}_{3}\,({D}_{3h},{H}_{0}+{H}_{3})=1-f\,({\lambda }_{3})/2$, with *f* (*λ*) = *λ* ^2^/(4*t* ^2^ + *λ* ^2^) and ${t}^{2}=2{t}_{1}^{2}+{t}_{2}^{2}$. Obviously, when ${\lambda }_{1}=2\sqrt{2}{\lambda }_{2}/3={\lambda }_{3}=\lambda$ the above three SSD are exactly coincident. It means there is no chance to distinguish these three kinds of symmetry breaking perturbations by SSD.

To distinguish three distinct symmetry breaking perturbations, we calculate the VSD straightforwardly according to its definition in Eq. (2) as

10 $${{\bf{S}}}_{1}\,({D}_{3h},{H}_{0}+{H}_{1})=\frac{1}{\sqrt{12}}(1,\sqrt{2},\sqrt{3}\sqrt{1-f({\lambda }_{1})},\sqrt{1-f({\lambda }_{1})},\sqrt{2}\sqrt{1-f({\lambda }_{1})},\sqrt{3}),$$

11 $${{\bf{S}}}_{2}\,({D}_{3h},{H}_{0}+{H}_{2})=\frac{1}{\sqrt{12}}(1,\sqrt{2-\frac{3}{2}f\,(\frac{2\sqrt{2}}{3}{\lambda }_{2})},\sqrt{3-\frac{3}{2}f\,(\frac{2\sqrt{2}}{3}{\lambda }_{2})},\,1,\,\sqrt{2-\frac{3}{2}f\,(\frac{2\sqrt{2}}{3}{\lambda }_{2})},\sqrt{3-\frac{3}{2}f\,(\frac{2\sqrt{2}}{3}{\lambda }_{2})}),$$

12 $${{\bf{S}}}_{3}\,({D}_{3h},{H}_{0}+{H}_{3})=\frac{1}{\sqrt{12}}(1,\,\sqrt{2-\frac{4}{3}f({\lambda }_{3})},\sqrt{3-\frac{5}{3}f\,({\lambda }_{3})},\sqrt{1-f({\lambda }_{3})},\sqrt{2-\frac{2}{3}f({\lambda }_{3})},\sqrt{3-\frac{4}{3}f({\lambda }_{3})}).$$

Firstly, for one single symmetry breaking perturbation, the VSD provides more information of symmetry nature than SSD. If we take **S**_**1**_(*D*_3*h*_, *H*_0_ + *H*_1_) as the example, the components for the conjugacy classes $\{E,2{C}_{3},3{\sigma }_{v}\}$ are invariant and the components for the conjugacy classes $\{3{C}_{2}^{^{\prime} },{\sigma }_{h},2{S}_{3}\}$ decreases, which means symmetry breaking perturbation *H*_1_ only breaks those symmetry transformations in the later conjugacy classes. Secondly, for distinct symmetry breaking perturbations, the VSDs are completely different from each other and thus the corresponding symmetry breaking perturbations are definitely distinguished, which is depicted in Fig. 1(c).

**Figure 2:**
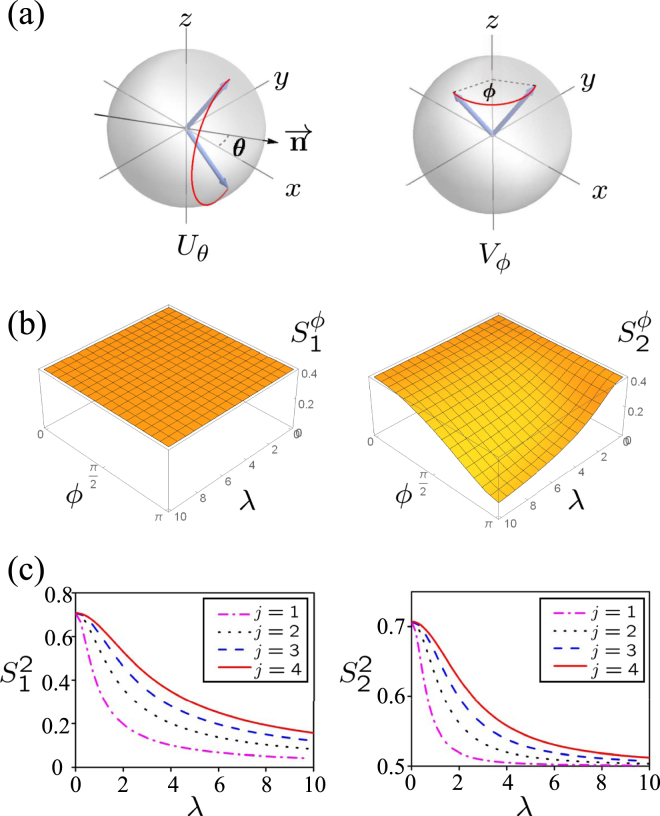
(**a**) Animation of symmetric transformations. Left and right plot denotes the 2-fold rotation along any axis in the *x* − *y* plane as *U*_*θ*_ = exp[−*i*(*J*_*x*_ cos *θ* + *J*_*y*_ sin *θ*)*π*], *θ* ∈ [0, 2*π*) and the continuous rotations along *z*-axis as *V*_*ϕ*_ = exp[−*iJ*_*z*_ *ϕ*], *ϕ* ∈ [0, 2*π*). The red solid curves represents the trajectories of the angular momentum under the symmetric transformations. Here, $\overrightarrow{{\bf{n}}}=(\cos \,[\theta ],\sin \,[\theta ])$ is the 2-fold rotation axis. (**b**) The components of VSDs under the first (left) and the second (right) symmetry breaking perturbations ${S}_{1}^{\varphi }$ and ${S}_{2}^{\varphi }$ versus *ϕ* and *λ*. Here, the angular quantum number is fixed as *j* = 4. (**c**) The components of VSDs under the first (left) and the second (right) symmetry breaking perturbations ${S}_{1}^{2}$ and ${S}_{2}^{2}$ versus *λ*. Here, the magenta dash-dotted line, the black dotted line, the blue dashed line and the red solid line respectively represent the angular momentum with quantum number *j* = 1, 2, 3, 4. Obviously, the VSDs for distinct symmetry breaking perturbations are obviously different.

The VSD not only works well for the finite group, but also for the infinite group which describes the continuous symmetry. The basic difference between them is that the infinite group possesses infinite number of conjugacy classes. In this sense, the VSD is regarded as a vector with infinite dimensions. For instance, we consider a physical system with angular momentum **J** described by the following Hamiltonian ${H}_{0}=\varepsilon {J}^{2}+\alpha {J}_{z}^{2}$, whose corresponding continuous symmetry group is $G=SU(1)\otimes {Z}_{2}$. The symmetric transformations include the 2-fold rotation along any axis in the *x* − *y* plane as ${U}_{\theta }=\exp \,[-i({J}_{x}\,\cos \,\theta +{J}_{y}\,\sin \,\theta )\pi ],\theta \in [0,\,2\pi )$ and the continuous rotations along *z*-axis as ${V}_{\varphi }=\exp \,[-i{J}_{z}\varphi ],\varphi \in [0,\,2\pi )$. The corresponding transformations are schematically illustrated in Fig. 2(a). It is easy to prove that all the 2-fold rotation transformations belong to the same conjugacy class. The rotations with respect to the *z* axis with rotation angle *ϕ* and −*ϕ* also belong to the same conjugacy class (see Appendix). Since the rotation angle *ϕ* can varies from 0 to *π*, obviously the number of conjugacy classes are infinite.

Two different perturbations are considered here *H*_1_ = *λαJ*_*z*_ and *H*_2_ = *λαJ*_*y*_, which respectively break *G* into subgroup *SU*(1) and *Z*_2_. In this sense, the coincident SSD are obtained as

13 $${{\mathscr{S}}}_{1}(G,{H}_{0}+{H}_{1})={{\mathscr{S}}}_{2}(G,{H}_{0}+{H}_{2})=1-{\lambda }^{2}/(2{\lambda }^{2}+\frac{8{j}^{2}+8j-6}{15}),$$

where *j* is the quantum number of the angular operator. The corresponding VSD are obtained as ${{\bf{S}}}_{1}\,(G,{H}_{0}+{H}_{1})=$ ${\int }_{0}^{\pi }d\varphi {S}_{1}^{\varphi }{{\bf{e}}}_{\varphi }+{S}_{1}^{2}{{\bf{e}}}_{2}$ and ${{\bf{S}}}_{2}(G,{H}_{0}+{H}_{2})={\int }_{0}^{\pi }d\varphi {S}_{2}^{\varphi }{{\bf{e}}}_{\varphi }+{S}_{2}^{2}{{\bf{e}}}_{2}$, where

14 $${S}_{1}^{\varphi }=\{\begin{array}{cc}\frac{1}{\sqrt{2\pi }}, & \varphi \ne 0,\pi \\ \frac{1}{2\sqrt{\pi }}, & \varphi =\pi \\ \frac{1}{2\sqrt{\pi }}, & \varphi =0\end{array},\,\,\,{S}_{2}^{\varphi }=\{\begin{array}{cc}\frac{1}{2\sqrt{\pi }}\sqrt{2-g(\lambda ,\theta )}, & \varphi \ne 0,\pi \\ \frac{1}{2\sqrt{\pi }}\sqrt{1-g(\lambda ,\frac{\pi }{2})}, & \varphi =\pi \\ \frac{1}{2\sqrt{\pi }}, & \varphi =0\end{array},$$

15 $${S}_{1}^{2}=\frac{1}{\sqrt{2}}\sqrt{1-g(\lambda ,\frac{\pi }{2})},\,\,{S}_{2}^{2}=\frac{1}{\sqrt{2}}\sqrt{1-\frac{1}{2}g(\lambda ,\frac{\pi }{2})},$$

with

16 $$g(\lambda ,\theta )=\frac{{\lambda }^{2}(1-\,\cos \,\varphi )}{{\lambda }^{2}+\frac{4{j}^{2}+4j-3}{15}}.$$

and **e**_*ϕ*_, **e**_2_ are the unit vectors with respect to the continuous rotations and 2-fold rotations respectively. The SSD ${{\mathscr{S}}}_{1}(G,{H}_{0}+{H}_{1})$ is still regarded as the square length of the VSD **S**_**1**_(*G*, *H*_0_ + *H*_1_) as

17 $$\begin{array}{ccc}{{\mathscr{S}}}_{1}(G,{H}_{0}+{H}_{1}) & \,= & \underset{0}{\overset{\pi }{\int }}d\varphi {|{S}_{1}^{\varphi }|}^{2}+{|{S}_{1}^{2}|}^{2}\\  & = & \frac{1}{2}+\frac{1}{2}(1-\frac{{\lambda }^{2}}{{\lambda }^{2}+\frac{4{j}^{2}+4j-3}{15}})\\  & = & 1-\frac{{\lambda }^{2}}{2{\lambda }^{2}+\frac{8{j}^{2}+8j-6}{15}}.\end{array}$$

The SSD ${{\mathscr{S}}}_{2}(G,{H}_{0}+{H}_{1})$ can be recovered from VSD S_2_(*G*, *H*_0_ + *H*_1_) in the same way. We can see from Fig. 2(b) and (c) that the first perturbation *H*_1_ only breaks the 2-fold rotations and keeps invariable under transformations in *SU*(1) while the second perturbation *H*_2_ breaks both continuous rotations and 2-fold rotations.

It should be indicated that the definition of VSD can be generalized to complicated cases, when the conjugacy class of the symmetry group is defined by multiple parameters. For instance, if the conjugacy class depends on *n* parameters such as *x*_1_, *x*_2_, …, *x*_*n*_, the VSD can be defined in a multiple integral form as ${\bf{S}}(G,H)=$ $\int \int \cdots \int d{x}_{1}d{x}_{2}\cdots d{x}_{n}S({x}_{1},{x}_{2},\cdots ,{x}_{n}){{\bf{e}}}_{{x}_{1},{x}_{2},\cdots ,{x}_{n}}$ with unit vector ${{\bf{e}}}_{{x}_{1},{x}_{2},\cdots ,{x}_{n}}$.

## Discussion

For a physical system with symmetry especially when it is broken somehow, the continuous quantitative description of the symmetry is required to characterize the physical system. In this work, we present the VSD instead of the SSD. Since the vector possesses not only the vector length which equals to the SSD, but also the components which provide information of symmetry degree on individual transformation. Therefore, the VSD provides more information of the symmetry nature.

For the symmetry breaking perturbations which break the original symmetry into different subgroups, VSD can be used to distinguish distinct symmetry breaking perturbations which just give the identical SSD values. Since the good properties of the VSD are inherited from the SSD, the VSD is feasible to identify other symmetry related effects and phenomenons, such as accidental degeneracy and spontaneous symmetry breaking. The evolution equation of the symmetry in the parameter space determines how the symmetry varies, which may have a deep relationship to the evolution of the quantum states under some time dependent perturbations such as the adiabatic process. The research of the symmetry degree will shed light on many physical applications such as the design of the artificial molecules, fabrication of Van de Vaals materials, classical and quantum critical phenomenons and so on.

## Methods

Before defining the symmetry degree with respect to the individual transformation, the asymmetry degree is defined in an intuitive way. For a physical system with Hamiltonian *H*, if we have the relation $R(g)H{R}^{\dagger }\,(g)=H$ where *R*(*g*) is a *d*-dimensional representation of *g* ∈ *G*, it means the Hamiltonian *H* is invariant under the transformation *g*. Obviously, *R*(*g*)*HR* ^†^ (*g*) ≠ *H* indicates that Hamiltonian *H* is no longer invariant under transformation *g*. Therefore, the deviation between *R*(*g*)*HR* ^†^ (*g*) and *H* can be used to measure the asymmetry of the physical system with respect to transformation *g*. Additionally, the Frobenius norm is introduced as the measure of such deviations.

In this sense, the asymmetry degree with respect to the individual transformation *g* is defined as

18 $$\begin{array}{c}{\mathscr{A}}(g,H)=\,\frac{1}{4{|\mathop{H}\limits^{ \sim }|}^{2}}{|R(g)\mathop{H}\limits^{ \sim }{R}^{\dagger }(g)-\mathop{H}\limits^{ \sim }|}^{2}\\ \,\,\,\,\,\,\,\,\,\,\,\,\,\,\,\,\,\,\,=\,\frac{1}{4{|\mathop{H}\limits^{ \sim }|}^{2}}Tr\,[{(R(g)\mathop{H}\limits^{ \sim }{R}^{\dagger }(g)-\mathop{H}\limits^{ \sim })}^{\dagger }(R(g)\mathop{H}\limits^{ \sim }{R}^{\dagger }\,(g)-\mathop{H}\limits^{ \sim })]\\ \,\,\,\,\,\,\,\,\,\,\,\,\,\,\,\,\,\,\,=\,\frac{1}{4{|\mathop{H}\limits^{ \sim }|}^{2}}Tr\,[2{\mathop{H}\limits^{ \sim }}^{2}-R(g)\mathop{H}\limits^{ \sim }{R}^{\dagger }(g)\mathop{H}\limits^{ \sim }-\mathop{H}\limits^{ \sim }R(g)\mathop{H}\limits^{ \sim }{R}^{\dagger }(g)]\\ \,\,\,\,\,\,\,\,\,\,\,\,\,\,\,\,\,\,\,=\,\frac{1}{4{|\mathop{H}\limits^{ \sim }|}^{2}}Tr\,[{(R(g)\mathop{H}\limits^{ \sim }-\mathop{H}\limits^{ \sim }R(g))}^{\dagger }(R(g)\mathop{H}\limits^{ \sim }-\mathop{H}\limits^{ \sim }R(g))]\\ \,\,\,\,\,\,\,\,\,\,\,\,\,\,\,\,\,\,\,=\,\frac{1}{4{|\mathop{H}\limits^{ \sim }|}^{2}}{|[R(g),\mathop{H}\limits^{ \sim }]|}^{2},\end{array}$$

where $4{|\mathop{H}\limits^{ \sim }|}^{2}$ is a naomalization factor, $\mathop{H}\limits^{ \sim }=H-{d}^{-1}Tr\{H\}$ is a re-biased Hamiltonian in order to make sure the asymmetry degree is zero-point energy independent.

Intuitively, we can define the symmetry degree with respect to the individual transformation *g* as

19 $$\begin{array}{c}{\mathscr{S}}(g,H)=1-{\mathscr{A}}(g,H)\\ \,\,\,\,\,\,\,\,\,\,\,\,\,\,\,\,\,\,=\,1-\frac{1}{4{|\mathop{H}\limits^{ \sim }|}^{2}}{|[R(g),\mathop{H}\limits^{ \sim }]|}^{2}\\ \,\,\,\,\,\,\,\,\,\,\,\,\,\,\,\,\,\,=\,\frac{1}{4{|\mathop{H}\limits^{ \sim }|}^{2}}(4{|\mathop{H}\limits^{ \sim }|}^{2}-{|[R(g),\mathop{H}\limits^{ \sim }]|}^{2})\\ \,\,\,\,\,\,\,\,\,\,\,\,\,\,\,\,\,\,=\,\frac{1}{4{|\mathop{H}\limits^{ \sim }|}^{2}}(4Tr[{\mathop{H}\limits^{ \sim }}^{2}]-Tr[({(R(g)\mathop{H}\limits^{ \sim }-\mathop{H}\limits^{ \sim }R(g))}^{\dagger }(R(g)\mathop{H}\limits^{ \sim }-\mathop{H}\limits^{ \sim }R(g)))])\\ \,\,\,\,\,\,\,\,\,\,\,\,\,\,\,\,\,\,=\,\frac{1}{4{|\mathop{H}\limits^{ \sim }|}^{2}}Tr[2{\mathop{H}\limits^{ \sim }}^{2}+R(g)\mathop{H}\limits^{ \sim }{R}^{\dagger }(g)\mathop{H}\limits^{ \sim }+HR(g)\mathop{H}\limits^{ \sim }{R}^{\dagger }(g)]\\ \,\,\,\,\,\,\,\,\,\,\,\,\,\,\,\,\,\,=\,\frac{1}{4{|\mathop{H}\limits^{ \sim }|}^{2}}Tr[{(R(g)\mathop{H}\limits^{ \sim }+\mathop{H}\limits^{ \sim }R(g))}^{\dagger }(R(g)\mathop{H}\limits^{ \sim }+\mathop{H}\limits^{ \sim }R(g))]\\ \,\,\,\,\,\,\,\,\,\,\,\,\,\,\,\,\,\,=\,\frac{1}{4{|\mathop{H}\limits^{ \sim }|}^{2}}{|\{R(g),\mathop{H}\limits^{ \sim }\}|}^{2},\end{array}$$

which is the origin of the definition of SSD and VSD in Eqs (1) and (2).

## Electronic supplementary material

Vector Form of Symmetry Degree
