# Supplementary material for: Vector Form of Symmetry Degree
Source: Sci Rep. 2017 Oct 11;7:12947. doi: 10.1038/s41598-017-13405-0 (PMC5636841; doi:10.1038/s41598-017-13405-0)
Supplement: Supplementary file 1 — Vector Form of Symmetry Degree [file 41598_2017_13405_MOESM1_ESM.pdf]

# Vector Form of Symmetry Degree

G. H. Dong,[1] Z. W. Zhang,[2] C. P. Sun,[1] and Z. R. Gong[2,1]

[1] *Beijing Computational Science Research Center, Beijing 100084, China*

[2] *College of Physics and Energy, Shenzhen University, Shenzhen, 518060, P. R. China*

We present details of the calculation of the VSDs for the physical system with angular momentum $J$ under two distinct symmetry breaking perturbations.

For the physical system with angular momentum $J$, we start with the symmetric Hamiltonian $H_0 = \epsilon J^2 + \alpha J_z^2$. The symmetric transformations include the 2-fold rotation along any axis in the $x-y$ plane as $U_\theta = \exp[-i(J_x \cos\theta + J_y \sin\theta)\pi], \theta \in [0, 2\pi)$ and the continuous rotations along $z$-axis as $V_\phi = \exp[-iJ_z\phi], \phi \in [0, 2\pi)$. Since we have following transformation relations as

$$U_{\theta'}^\dagger U_\theta U_{\theta'} = U_\theta, \tag{1}$$

$$V_{\phi'}^\dagger V_\phi V_{\phi'} = V_\phi, \tag{2}$$

$$V_\phi^\dagger U_\theta V_\phi = U_{\theta+\phi}, \tag{3}$$

$$U_\theta^\dagger V_\phi U_\theta = V_{-\phi}, \tag{4}$$

all the 2-fold rotation transformations belong to the same conjugacy class, and the rotations with respect to the $z$ axis with rotation angle $\phi$ and $-\phi$ also belong to the same conjugacy class.

The Hamiltonian with the first kind of symmetry breaking perturbation reads

$$H_0 + H_1 = J^2 + \alpha J_z^2 + \lambda\alpha J_z. \tag{5}$$

Then the biased Hamiltonian is

$$\tilde{H}_0 + \tilde{H}_1 = \alpha J_z^2 + \lambda\alpha J_z - \frac{\alpha}{3} j(j+1)(2j+1) I_{(2j+1)\times(2j+1)}, \tag{6}$$

where $j$ is the angular quantum number and $I_{(2j+1)\times(2j+1)}$ is the $(2j+1)\times(2j+1)$ identity matrix. In this sense, the following Frobenius norms are obtained as

$$\left|\left\{e^{-iJ_z\phi}, \left(\tilde{H}_0 + \tilde{H}_1\right)\right\}\right|^2 = 4\mathrm{Tr}\left(\tilde{H}_0 + \tilde{H}_1\right)^2, \tag{7}$$

$$\left|\left\{e^{-i(J_x\cos\theta + J_y\sin\theta)\pi}, \left(\tilde{H}_0 + \tilde{H}_1\right)\right\}\right|^2 = 4\mathrm{Tr}\left(\tilde{H}_0 + \tilde{H}_1\right)^2 - 4\lambda\alpha\mathrm{Tr}\left(J_z(\tilde{H}_0 + \tilde{H}_1)\right), \tag{8}$$

with

$$\mathrm{Tr}\left(\tilde{H}_0 + \tilde{H}_1\right)^2 = j(j+1)(2j+1)\frac{\alpha^2}{3}\left(\lambda^2 + \frac{4j^2 + 4j - 3}{15}\right), \tag{9}$$

$$\mathrm{Tr}\left(J_z(\tilde{H}_0 + \tilde{H}_1)\right) = \frac{\lambda\alpha}{3} j(j+1)(2j+1). \tag{10}$$

Therefore, the components of the first VSD $\mathbf{S}_1(G, H_0 + H_1)$ respectively are

$$S_1^{\phi=0} = \sqrt{\frac{1}{4\pi}\frac{\left|\left\{e^{-iJ_z\phi},\left(\tilde{H}_0+\tilde{H}_1\right)\right\}\right|^2_{\phi=0}}{4\mathrm{Tr}\left(\tilde{H}_0+\tilde{H}_1\right)^2}} = \frac{1}{2\sqrt{\pi}}, \tag{11}$$

$$S_1^{\phi\in(0,\pi)} = \sqrt{\frac{1}{4\pi}}\sqrt{\frac{\left|\left\{e^{-iJ_z\phi},\left(\tilde{H}_0+\tilde{H}_1\right)\right\}\right|^2_{\phi\in(0,\pi)}}{4\mathrm{Tr}\left(\tilde{H}_0+\tilde{H}_1\right)^2}+\frac{\left|\left\{e^{iJ_z\phi},\left(\tilde{H}_0+\tilde{H}_1\right)\right\}\right|^2_{\phi\in(0,\pi)}}{4\mathrm{Tr}\left(\tilde{H}_0+\tilde{H}_1\right)^2}} = \frac{1}{\sqrt{2\pi}}, \tag{12}$$

$$S_1^{\phi=\pi} = \sqrt{\frac{1}{4\pi}\frac{\left|\left\{e^{-iJ_z\phi},\left(\tilde{H}_0+\tilde{H}_1\right)\right\}\right|^2_{\phi=\pi}}{4\mathrm{Tr}\left(\tilde{H}_0+\tilde{H}_1\right)^2}} = \frac{1}{2\sqrt{\pi}}, \tag{13}$$

$$S_1^{\theta} = \sqrt{\frac{1}{4\pi}\int_0^{2\pi}\frac{\left|\left\{e^{-i(J_x\cos\theta+J_y\sin\theta)\pi},\left(\tilde{H}_0+\tilde{H}_1\right)\right\}\right|^2}{4\left|\tilde{H}_0+\tilde{H}_1\right|^2}d\theta} = \frac{1}{\sqrt{2}}\sqrt{1-\frac{\lambda^2}{\lambda^2+\frac{4j^2+4j-3}{15}}}. \tag{14}$$

For the second symmetry breaking perturbation, the total Hamiltonian reads

$$H_0+H_1 = J^2+\alpha J_z^2+\lambda\alpha J_y \tag{15}$$

with the biased Hamiltonian

$$\tilde{H}_0+\tilde{H}_2 = \alpha J_z^2+\lambda\alpha J_y-\frac{\alpha}{3}j(j+1)(2j+1)I_{(2j+1)\times(2j+1)}. \tag{16}$$

The Frobenius norms for the VSD are straightforwardly calculated as

$$\left|\left\{e^{-iJ_z\phi},\left(\tilde{H}_0+\tilde{H}_2\right)\right\}\right|^2 = 4\left|\tilde{H}_0+\tilde{H}_2\right|^2-2\lambda^2\alpha^2\left(1-\cos\phi\right)\mathrm{Tr}J_z^2, \tag{17}$$

$$\left|\left\{e^{-i(J_x\cos\theta+J_y\sin\theta)\pi},\left(\tilde{H}_0+\tilde{H}_2\right)\right\}\right|^2 = 4\left|\tilde{H}_0+\tilde{H}_2\right|^2-2\lambda^2\alpha^2\left(1-\cos 2\theta\right)\mathrm{Tr}J_z^2, \tag{18}$$

with

$$\left|\tilde{H}_0+\tilde{H}_2\right|^2 = j(j+1)(2j+1)\frac{\alpha^2}{3}\left(\lambda^2+\frac{4j^2+4j-3}{15}\right), \tag{19}$$

$$\mathrm{Tr}J_z^2 = \frac{1}{3}j(j+1)(2j+1). \tag{20}$$

Therefore, the components of the second VSD $\mathbf{S}_2(G,H_0+H_2)$ respectively are

$$
\begin{aligned}
S_2^{\phi=0} &= \sqrt{\frac{1}{4\pi}\frac{\left|\left\{e^{-iJ_z\phi},\left(\tilde{H}_0+\tilde{H}_2\right)\right\}\right|^2_{\phi=0}}{4\mathrm{Tr}\left(\tilde{H}_0+\tilde{H}_2\right)^2}} \\
&= \frac{1}{2\sqrt{\pi}}, \qquad (21)\\
S_2^{\phi\in(0,\pi)} &= \sqrt{\frac{1}{4\pi}\frac{\left|\left\{e^{-iJ_z\phi},\left(\tilde{H}_0+\tilde{H}_2\right)\right\}\right|^2_{\phi\in(0,\pi)}}{4\mathrm{Tr}\left(\tilde{H}_0+\tilde{H}_2\right)^2}+\frac{1}{4\pi}\frac{\left|\left\{e^{iJ_z\phi},\left(\tilde{H}_0+\tilde{H}_2\right)\right\}\right|^2_{\phi\in(0,\pi)}}{4\mathrm{Tr}\left(\tilde{H}_0+\tilde{H}_2\right)^2}} \\
&= \frac{1}{2\sqrt{\pi}}\sqrt{2-\frac{\lambda^2\left(1-\cos\phi\right)}{\lambda^2+\frac{4j^2+4j-3}{15}}}, \qquad (22)\\
S_2^{\phi=\pi} &= \sqrt{\frac{1}{4\pi}\frac{\left|\left\{e^{-iJ_z\phi},\left(\tilde{H}_0+\tilde{H}_2\right)\right\}\right|^2_{\phi=\pi}}{4\mathrm{Tr}\left(\tilde{H}_0+\tilde{H}_2\right)^2}} \\
&= \frac{1}{2\sqrt{\pi}}\sqrt{1-\frac{\lambda^2}{\lambda^2+\frac{4j^2+4j-3}{15}}}, \qquad (23)\\
S_2^{\theta} &= \sqrt{\frac{1}{4\pi}\int_0^{2\pi}\frac{\left|\left\{e^{-i(J_x\cos\theta+J_y\sin\theta)\pi},\left(\tilde{H}_0+\tilde{H}_2\right)\right\}\right|^2}{4\left|\tilde{H}_0+\tilde{H}_2\right|^2}d\theta} \\
&= \sqrt{\frac{1}{4\pi}\int_0^{2\pi}\left(1-\frac{\lambda^2\left(1-\cos2\theta\right)}{2\left[\lambda^2+\frac{4j^2+4j-3}{15}\right]}\right)d\theta} \\
&= \frac{1}{2}\sqrt{2-\frac{\lambda^2}{\lambda^2+\frac{4j^2+4j-3}{15}}}. \qquad (24)
\end{aligned}
$$
